# miR-526b targets 3′ UTR of *MMP1* mRNA

**DOI:** 10.1038/emm.2015.52

**Published:** 2015-08-21

**Authors:** Kyu-Han Kim, Ji-Yong Jung, Eui Dong Son, Dong Wook Shin, Minsoo Noh, Tae Ryong Lee

**Affiliations:** 1 Bioscience Research Institute, AmorePacific Corporation R&D Center, Gyeonggi-do, Republic of Korea; 2 Natural Products Research Institute, College of Pharmacy, Seoul National University, Seoul, Republic of Korea

## Abstract

Regulation of matrix metalloproteinases (MMPs) is important for many physiological processes involving cancers, inflammation, tissue remodeling and skin aging. Here, we report the novel finding that the expression of *MMP1* mRNA is downregulated by the overexpression of miR-526b which is a member of chromosome 19 microRNA cluster (C19MC). Our analysis using reporter constructs containing the 3′ untranslated region (3′ UTR) of *MMP1* and its mutant form showed that the region from 377–383 in the 3′ UTR of *MMP1* is critical for targeting by miR-526b. In addition, the expression pattern of miR-526b and *MMP1* mRNA showed reverse relation between adult dermal and neonatal fibroblasts. We show for the first time that miR-526b, an miRNA belonging to C19MC, can target the 377–383 region of the *MMP1* 3′ UTR.

## Introduction

Matrix metalloproteinases (MMPs) are a family of zinc-dependent proteases that have important roles in several normal physiological processes, such as inflammation, tissue remodeling, skin aging, embryonic development and placental functions, as well as in disease conditions, such as cancer. MMPs can be classified into collagenases, gelatinases, stromelysins, membrane-type MMPs and other MMPs, according to their structure and substrate specificities.^1, 2, 3^ In skin, fibroblasts produce several MMPs, including interstitial collagenase (collagenase-1, MMP1), stromelysin-1 (MMP3) and a 72-kDa gelatinase (MMP2). MMP1 is a member of the collagenase subgroup that causes proteolytic degradation of type-1 and type-3 collagens as well as elastic fibers and is thought to be related to several pathological conditions, including dermal photoaging, cutaneous ulcer and wrinkles.^4, 5^

MicroRNAs (miRNAs) are noncoding small RNAs ~21–23 nucleotides long that regulate mRNA expression. miRNA is processed from a longer transcript, primary miRNA, into mature miRNA through a series of post-transcriptional biogenesis steps. Then if the mature miRNA is loaded onto an argonaute-containing RNA-induced silencing complex, this complex will target specific 3′ untranslated region (3′ UTR) regions of mRNA, resulting in the inactivation of the mRNA through either degradation or translational repression.^6^

Post-transcriptional regulation by miRNAs in skin is a critical process, as evidenced by recent studies on miRNAs.^7, 8, 9^ Many studies have focused on the identification of miRNA-target mRNA pairs, whereas little attention has been paid to miRNAs targeting *MMP1*. In the present study, we showed that miR-526b, an miRNA belonging to chromosome 19 miRNA cluster (C19MC), targets *MMP1* through the binding to the specific 3′ UTR region of the *MMP1* mRNA.

## Materials and Methods

### Cell culture and treatment

Dermal fibroblasts from human neonatal and adult foreskin were obtained from Lonza (Walkersville, MD, USA) and maintained in Dulbecco′s modified Eagle′s medium supplemented with 1% penicillin/streptomycin and 10% heat-inactivated fetal bovine serum (Gibco, Carlsbad, CA, USA). The fibroblasts were cultured until they were 90% confluent before being passaged. WM-115 human melanoma cells were obtained from American Type Culture Collection (ATCC, Manassas, VA, USA) and maintained in Eagle's Minimum Essential Medium supplemented with 1% penicillin/streptomycin and 10% heat-inactivated fetal bovine serum. HeLa human cervical cancer cells were obtained from Korean Cell Line Bank (Seoul, Republic of Korea) and maintained in Dulbecco′s modified Eagle′s medium supplemented with 1% penicillin/streptomycin and 10% heat-inactivated fetal bovine serum. The JAR human placenta choriocarcinoma cell line was obtained from Korean Cell Line Bank and maintained in Roswell Park Memorial Institute medium supplemented with 1% penicillin/streptomycin and 10% heat-inactivated fetal bovine serum. All cells were grown at 37 °C with 5% CO_2_.

To determine whether miR-526b is regulated by epigenetic mechanisms, HeLa cells were treated with 5-μM 2′-deoxy-5-azacytidine (5′aza, Sigma, #A3656, St Louis, MO, USA) for 3 days. Subsequently, 1-μM trichostatin A (TSA, Sigma, Cat #T1952) was added directly to the medium for 1 day. For the analysis of *MMP1* regulation by miR-526b, fibroblasts were transfected with hsa-miR-526b and hsa-miR-1248 mimics (Dharmacon, Lafayette, CO, USA) using RNAiMAX Lipofectamine reagents (Invitrogen, Carlsbad, CA, USA) according to the manufacturer's instructions. We used miRIDIAN microRNA Mimic Negative Control #1 (Dharmacon, Cat # CN-001000-01-05) as the negative control (N.C.) mimic.

### RNA extraction and quantitative real-time PCR

Total RNA, including small RNA, was isolated using TRIzol reagent (Invitrogen) according to the manufacturer's instructions. To examine the expression of *MMP* mRNA, cDNAs were synthesized from the total RNA using the Superscript Reverse Transcriptase (RT) II kit (Invitrogen) according to the manufacturer's instructions. Quantitative mRNA measurements in each sample were carried out using the TaqMan Universal PCR Master Mix (Applied Biosystems, Foster City, CA, USA). The cDNA samples were analyzed for the following mRNA using the respective probes: *MMP1*, Hs00233958_m1; *MMP3*, Hs00968308_m1; *MMP8*, Hs01029057_m1; and *MMP13*, Hs00233992_m1. Human *GAPDH* (4333764 F, Applied Biosystems) was also amplified to normalize variations in the cDNA quantities from different samples. To examine the expression of miRNAs, the total RNA was reverse-transcribed using the TaqMan MicroRNA Reverse Transcription Kit (Applied Biosystems) according to the manufacturer's instructions and subjected to the TaqMan miRNA Assay (Applied Biosystems) using respective probes: miR-526b, 002382; and miR-1248, 002870. U6 snRNA (001093, Applied Biosystems) was used to normalize the miR-526b expression readings. Quantitative real-time PCR (RT-qPCR) analysis was carried out on an ABI 7500 Fast System (Applied Biosystems).

### Western blot

Adult dermal fibroblasts were transfected with miR-526b, miR-1248, or N.C. mimic. The next day, medium was removed, and cells were again placed in serum-free medium for 2 days. Conditioned mediums were subjected to SDS–polyacrylamide gel electrophoresis. After transferring the proteins and blocking membranes, the membranes were incubated overnight at 4 °C with anti-MMP-1 antibody (Santa Cruz Biotechnology, Santa Cruz, CA, USA). All protein bands were detected using an ECL chemiluminescence system (Amersham Pharmacia Biotech, Piscataway, NJ, USA).

### WST-1 assay

Cell viability was measured in 96-well plates using 2-(4-iodophenyl)-3-(4-nitrophenyl)-5-(2,4-disulfophenyl)-2H-tetrazolium monosodium salt (WST-1) reagent (Takara Bio, Shiga, Japan). After adding 10 μl of WST-1 reagent to each well for 45 min at 37 °C, the reduction rate of tetrazolium salts was estimated by the increase in OD_450_.

### Plasmid constructions

For the functional analysis of miR-526b and miR-1248, partial segments of the 3′ UTR of *MMP1* mRNA containing the predicted miR-526b- & miR-1248-binding sequences were amplified with PCR using the following primer set flanking the *Spe*I and *Hin*dIII restriction enzyme sites: for the wild type, CTAGACTAGTTTTGAATGGAAAACACATGGTG, CCCAAGCTTAAACAAGGTTGACTTTATTCCAAA. The PCR products were cloned into the pMIR-REPORT Firefly Luciferase reporter vector (Ambion, Austin, TX, USA). Site-directed mutagenesis was performed by overlapping PCR using the following primers: TTGCCGGAGGAAAAGCAGCTCCCTCACACATGTGCAGTCACTGG, CCAGTGACTGCACATGTGTGAGGGAGCTGCTTTTCCTCCGGCAA (the mutated sequences are underlined.)

### Luciferase assay

JAR placenta cells and HeLa cells were plated in 12-well plates and transfected with a reporter vector for the 3′UTR of *MMP1* mRNA using Lipofectamine 2000 (Invitrogen). All cells were co-transfected with a β-galactosidase expression vector (pSV-β-galactosidase) to normalize the transfection efficiency. The luciferase activity was measured using a Luciferase Assay Kit (Promega, Madison, WI, USA), and β-galactosidase activity was measured using the β-Galactosidase Enzyme Assay System (Promega) according to the manufacturer's protocol.

### Statistical analysis

All results are expressed as the mean±s.e.m. from at least three independent experiments. The difference between groups was calculated using the Student's *t*-test. Differences with a *P*<0.05 were considered statistically significant.

## Results

### Overexpression of miR-526b decreased the expression of *MMP1 mRNA*

To identify miRNAs that affect *MMP1 mRNA* expression, we used two computational prediction programs, targetscan (http://www.targetscan.org/), and microcosm (http://www.ebi.ac.uk/enright-srv/microcosm/htdocs/targets/v5/). Among the top five candidates predicted from microcosm algorithm, miR-526b has the highest score in the prediction by targetscan algorithm. Therefore, we suggested that miR-526b could be potent target of *MMP1* 3′ UTR. To compare the effect of miR-526b on targeting *MMP1*, we also tested the effect of miR-1248 which was only predicted by targetscan algorithm.

**Figure 1:**
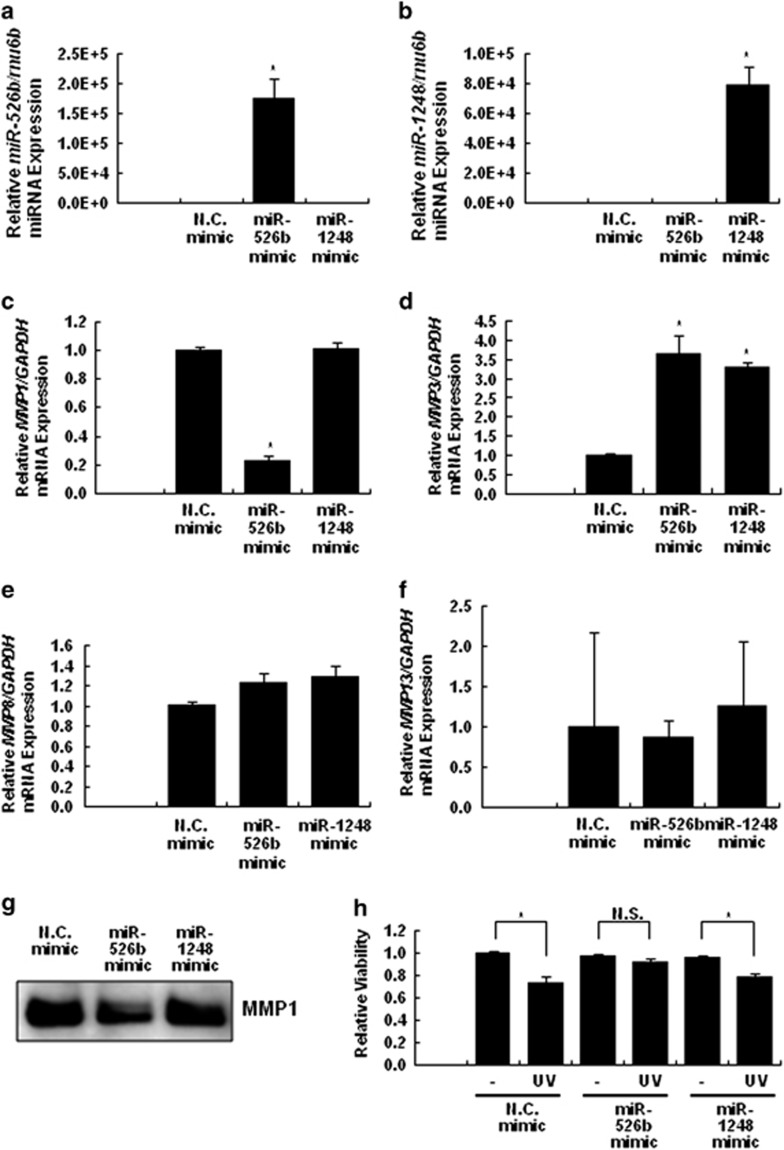
Downregulation of *MMP1* mRNA expression by miR-526b. Human adult primary fibroblasts were transfected with 20-μM miR-526b mimic, the miR-1248 mimic or N.C. mimic. The expression of (**a**) miR-526b and (**b**) miR-1248 were analyzed using TaqMan miRNA RT-qPCR and normalized to the expression of rnu6b (U6). The expression of (**c**) *MMP1*, (**d**) *MMP3*, (**e**) *MMP8* and (**f**) *MMP13* mRNA were analyzed using RT-qPCR and normalized to the expression of *GAPDH* mRNA. The data are shown as the mean±s.e.m. of at least three independent experiments (*P<0.05 compared with N.C. treated cells). (**g**) Proteins of MMP-1 expressed in the serum-free conditioned media from fibroblasts transfected with miR-526b, miR-1248 or N.C. mimic were analyzed by western blot. (**h**) Fibroblasts transfected with miR-526b, miR-1248 or N.C. mimic were irradiated with UVB light at 60 mJ cm^−2^. After 2 days, the cell viability was measured by WST-1 assay.

To evaluate whether miR-526b and miR-1248 are capable of *MMP1* mRNA regulation, we transfected human adult dermal fibroblasts, which express *MMP1* mRNA, with miR-526b or miR-1248 mimic (Figures 1a and b). As shown in Figure 1c, the decrease in *MMP1* expression was observed in fibroblasts overexpressing miR-526b but not miR-1248. Moreover, the protein level of MMP1 was reduced by the overexpression of miR-526b but not miR-1248 (Figure 1g). However, ectopic transfection with miR-526b or miR-1248 mimic did not reduce the expression of the other MMPs, including *MMP8* and *MMP13* (Figures 1e and f). Additionally, we observed that *MMP3 mRNA* was increased by miR-526b and miR-1248 (Figure 1d). We suggested that these miRNAs could regulate a repressor of *MMP3 mRNA*. However, because this was not our primary focus in this research, we did not further investigate how these miRNAs induced *MMP3* expression. Additionally, we observed that miR-526b has the protective effect on fibroblasts against UV exposure. We performed the WST1 assay to measure the cell viabilities of fibroblasts which were transfected miR-526b, miR-1248 or N.C. mimic after UV exposure. As shown in Figure 1h, miR-526b following the *MMP1* downregulation has the protective function under the UV exposure.

### The expression pattern of miR-526b is inversely proportional to that of *MMP1 mRNA*

**Figure 2:**
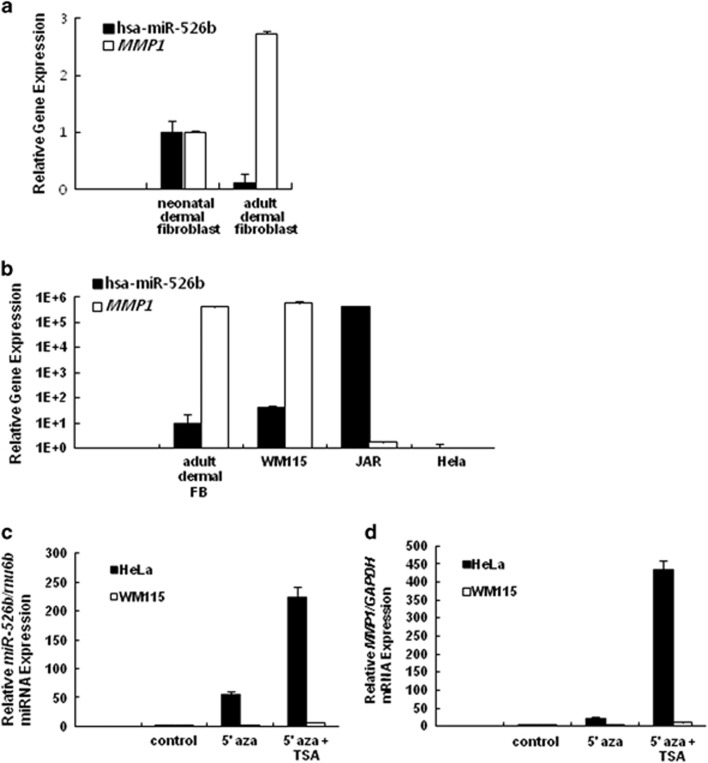
Expression pattern of miR-526b and *MMP1* mRNA. (**a**) The expression of *MMP1* mRNA, normalized to the expression of *GAPDH*, and mature miR-526b, normalized to the expression of U6, in neonatal and adult dermal fibroblasts were analyzed using RT-qPCR. The value obtained from neonatal dermal fibroblasts was set as 1. (**b**) The expression of *MMP1 mRNA*, normalized to the expression of GAPDH, and mature miR-526b, normalized to the expression of U6, in adult dermal fibroblasts, WM115, JAR and HeLa cells were analyzed using RT-qPCR. The value of *MMP1 mRNA* and miR-526b miRNA expression obtained from HeLa cells were set as 1. The *y* axis represents the expression level in log scale. (**c** and **d**) HeLa cells and WM115 cells were treated with 5 μM 5′aza for 3 days and then treated with 1 μM TSA for 1 day. The total RNA was extracted and analyzed for mature miR-526b expression (**c**) and *MMP1* expression (**d**).

To investigate the expression pattern of miR-526b and *MMP1* mRNA, we examined the basal expression levels of miR-526b and *MMP1* mRNA in fibroblast cells. As shown in Figure 2a, neonatal dermal fibroblasts expressed lower levels of *MMP1* mRNA compared with adult dermal fibroblasts, as previously reported.^10, 11^ In contrast, the expression of miR-526b in neonatal fibroblasts is higher than in adult fibroblasts (Figure 2a), indicating that miR-526b expression is inversely proportional to *MMP1* expression in fibroblast cells. In addition, we observed that in the JAR placenta cell line *MMP1* mRNA is expressed at a very low level while miR-526b is strongly expressed, while dermal fibroblasts, WM115, showed high expression level of *MMP1* but low level of miR-526b, implying that these cells showed the reverse relation of expression between miR-526b miRNA and MMP1 mRNA (Figure 2b). However, HeLa cells showed the low expression of both miR-526b and *MMP1*. We suggested that the transcription of both miR-526b and *MMP1* in HeLa cells are suppressed by the epigenetic control. It was reported that in many cells C19MC is expressed at a low level due to DNA methylation at the CpG island.^12^ To determine whether miR-526b and MMP1 in HeLa cells are also regulated by epigenetic mechanisms similar to the miRNAs involved in C19MC, 5′aza, a DNA methylation inhibitor, and TSA, a histone deacetylase inhibitor were treated in HeLa cells. Additionally, we treated WM115 cells which used as N.C. As shown in Figures 2c and d, 5′aza and TSA treatment strongly increased the expression of miR-526b and *MMP1* in HeLa cells but not WM115 cells. We suggested that *MMP1* and *miR-526b* are inversely regulated but both genes are sometimes suppressed in some cells through the epigenetic control.

### miR-526b targets the 3′ UTR of MMP1 mRNA

**Figure 3:**
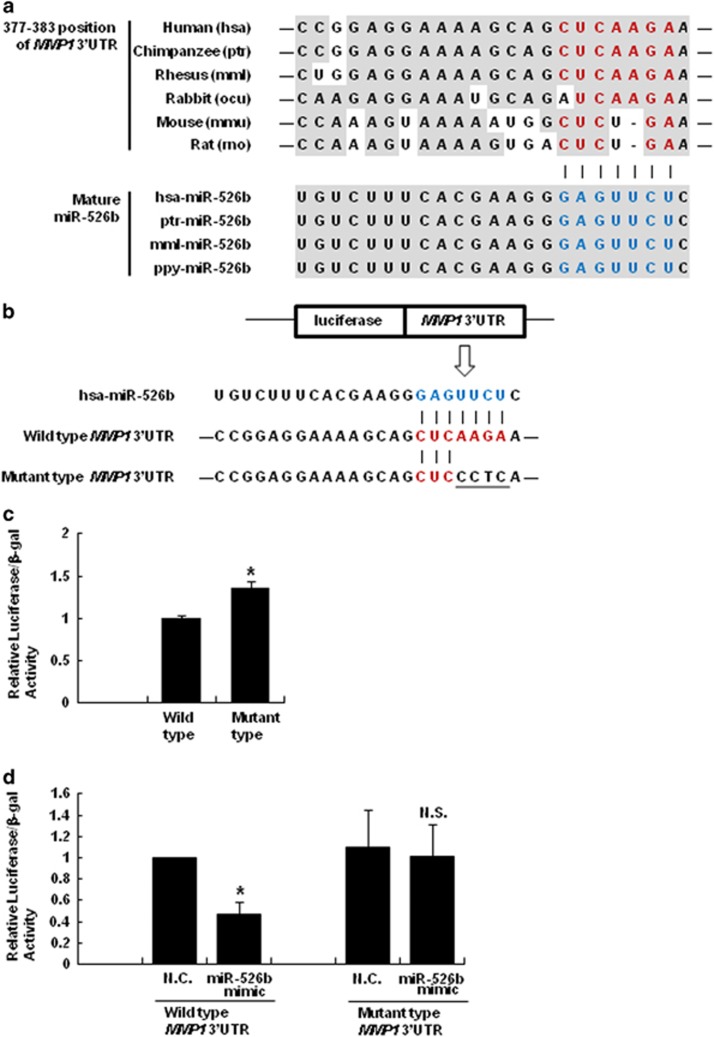
Direct targeting *MMP1* 3′ UTR of miR-526b. (**a**) The nucleotide resolution of the predicted target sites (377–383 position) of the *MMP1* 3′ UTR and mature miR-526b sequence: the seed sequence (blue letters); the target sequence (red letters); and the evolutionarily conserved regions (gray boxes). All of the sequences for mature miR-526b from human (hsa), chimpanzee (ptr), rhesus monkeys (mml) and orangutan (ppy) were perfectly conserved. The predicted target sequences of the *MMP1* 3′ UTR were only conserved in primates, including humans, chimpanzees and rhesus monkeys. (**b**) A sketch of the construction and sequence alignment of the wild-type and mutant *MMP1* 3′ UTR with hsa-miR-526b is shown. The mutated sequences are underlined. (**c**) The wild-type or mutant *MMP1* 3′ UTR reporter vector was transfected into JAR cells. The luciferase and β-galactosidase activities were measured after 72 h. The luciferase activities were normalized to the β-galactosidase activities. (**d**) The wild-type or mutant *MMP1* 3′ UTR reporter vector was co-transfected into the HeLa cells with either the N.C. mimic or miR-526b mimic. The luciferase and β-galactosidase activities were measured after 72 h. The luciferase activities were normalized to the β-galactosidase activities. The data are shown as the mean±s.e.m. of at least three independent experiments (*P<0.05).

Next, we verified whether *MMP1* mRNA is indeed a direct target for miR-526b. A single binding site on the 3′ UTR of *MMP1* mRNA at nucleotides 377–383 was predicted by the TargetScan algorithm (GeneBank Accession Numbers NM_002421). This predicted binding site is conserved only in primates, including humans, chimpanzees and rhesus monkeys (Figure 3a).

To validate the direct binding site of miR-526b in the 3′ UTR of *MMP1* mRNA, we performed a 3′UTR reporter-binding assay. The 3′ UTR region of *MMP1* mRNA was cloned into a luciferase reporter plasmid and subsequently mutated the predicted sequences bound by miR-526b (Figure 3b). And we transfected into JAR placenta cells, which express miR-526b with these luciferase vectors. As illustrated in Figure 3c, the luciferase activity in mutant type was higher than the one in wild type, indicating that the position of 377–383 on 3′ UTR of *MMP1* mRNA mediates the targeting of miR-526b. In order to confirm this result, we co-transfected into HeLa cells with 3′ UTR reporter vector, together with miR-526b mimic or their respective N.C. mimic. As shown in Figure 3d, the overexpression of miR-526b induced the reduced activity of *MMP1* 3′ UTR luciferase. However, the reduced activity by miR-526b was not observed with mutant type of *MMP1* 3′ UTR reporter vector containing the mutated sequences in predicted miR-526b binding site.

## Discussion

miR-526b belongs to the C19MC cluster which is the largest human miRNA gene cluster discovered to date and is composed of 46 repeated miRNA genes spanning ~100 kb on the human chromosome 19 q13.4 region. Previous studies have shown that C19MC is specifically expressed from the paternally inherited chromosome in placenta through epigenetic control in the CpG-rich region. Although the mechanisms for the generation and regulation of C19MC expression are relatively well known, none of the previously conducted studies have characterized the target of the individual miRNAs in C19MC.^12, 13, 14, 15^ In the present report, we show for the first time that miR-526b of the C19MC cluster targets the 3′ UTR of *MMP1* mRNA.

The C19MC cluster is only observed in primate chromosomes. Our results also show that the matching of miR-526b and *MMP1* mRNA is a primate-specific feature. Although four orthologs of miR-526b gene listed in the miRBase database have perfect similarity in their mature sequences, the complementary sequences of the *MMP1* 3′ UTR (red letters in Figure 3a) targeted by the seed sequences of miR-526b (blue letters in Figure 3a) are conserved exclusively in primates including humans, chimpanzees and rhesus monkeys, implying that the regulation of *MMP1* mRNA by miR-526b could contribute to primate-specific pathological functions. Moreover, we monitored other miRNAs clustered in C19MC could have the function of targeting *MMP1* mRNA. According to the prediction from TargetScan algorithm, hsa-miR-519c-5p, hsa-miR-519b-5p, hsa-miR-518d-5p, hsa-miR-526a and hsa-miR-520c have the same seed sequences complementary to 89–95 positions of 3′ UTR of *MMP1* mRNA. These sequences are so highly similar to the one of hsa-miR-526b that these miRNA would target *MMP1* mRNA. Although further studies are required to confirm this suggestion, we imply that C19MC could have a universal function for the suppression of *MMP1* mRNA expression in primates.

It was recently reported that hsa-miR-526b significantly suppresses the non-small cell lung cancer growth *in vitro* and *in vivo* by targeting Ku80 and its expression level is found to be frequently decreased in non-small cell lung cancer tissues.^16^ Meanwhile, MMP1 was known to be a critical factor involved in tumorigenesis.^17, 18^ In lung cancers, MMP1 is associated with an increased risk for lung cancer.^19^ In addition, mice deficient in MMP1a hamper tumor progression.^20^ Therefore, we could suggest that the regulation of MMP1 expression by miR-526b could be important in the regulation of lung cancer progression.

In summary, our data indicated that MMP1 expression is regulated by miR-526b. Seed sequences in miR-526b mRNA is bound to the position 377–383 of *MMP1* 3′ UTR, which is primate-specific phenomenon. These finding facilitate a better understanding of MMP1 regulation and the function of C19MC.
